# Testing Multiple Coordination Constraints with a Novel Bimanual Visuomotor Task

**DOI:** 10.1371/journal.pone.0023619

**Published:** 2011-08-17

**Authors:** Helene M. Sisti, Monique Geurts, René Clerckx, Jolien Gooijers, James P. Coxon, Marcus H. Heitger, Karen Caeyenberghs, Iseult A. M. Beets, Leen Serbruyns, Stephan P. Swinnen

**Affiliations:** Motor Control Laboratory, Research Center of Movement Control and Neuroplasticity, Department of Biomedical Kinesiology, Group Biomedical Sciences, Katholieke Universiteit Leuven, Leuven, Belgium; The University of Western Ontario, Canada

## Abstract

The acquisition of a new bimanual skill depends on several motor coordination constraints. To date, coordination constraints have often been tested relatively independently of one another, particularly with respect to isofrequency and multifrequency rhythms. Here, we used a new paradigm to test the interaction of multiple coordination constraints. Coordination constraints that were tested included temporal complexity, directionality, muscle grouping, and hand dominance. Twenty-two healthy young adults performed a bimanual dial rotation task that required left and right hand coordination to track a moving target on a computer monitor. Two groups were compared, either with or without four days of practice with augmented visual feedback. Four directional patterns were tested such that both hands moved either rightward (clockwise), leftward (counterclockwise), inward or outward relative to each other. Seven frequency ratios (3∶1, 2∶1, 3∶2, 1∶1, 2∶3. 1∶2, 1∶3) between the left and right hand were introduced. As expected, isofrequency patterns (1∶1) were performed more successfully than multifrequency patterns (non 1∶1). In addition, performance was more accurate when participants were required to move faster with the dominant right hand (1∶3, 1∶2 and 2∶3) than with the non-dominant left hand (3∶1, 2∶1, 3∶2). Interestingly, performance deteriorated as the relative angular velocity between the two hands increased, regardless of whether the required frequency ratio was an integer or non-integer. This contrasted with previous finger tapping research where the integer ratios generally led to less error than the non-integer ratios. We suggest that this is due to the different movement topologies that are required of each paradigm. Overall, we found that this visuomotor task was useful for testing the interaction of multiple coordination constraints as well as the release from these constraints with practice in the presence of augmented visual feedback.

## Introduction

Throughout our daily activities, both hands work together to achieve a broad range of tasks. As such, advancing our understanding of bimanual coordination principles has important implications for both healthy and clinical populations [1], [2], [3], [4]. Across species, the limbs have an innate tendency to move in an isochronous manner [2], [5]. While isofrequency patterns are the most stable, humans are capable of coordinating their left and right hands with a rich variety of rhythmic complexity, sometimes called polyrhythms [6], [7], [8], [9], [10], [11]. The study of isofrequency patterns and polyrhythms represent two major branches of bimanual coordination research with a long history [12], [13]. Interestingly, these two categories are often investigated independently of one another [3], [9].

Research focused on coordination of multifrequency rhythms typically uses the finger tapping task [3], [9]. However, experiments addressing stability of isofrequency rhythms usually include wrist and (fore)arm motions [1]. While many multifrequency patterns can be learned, finger tapping studies have revealed that some patterns are acquired more rapidly than others [8], [9]. For instance, integer ratios are easier to perform than non-integer ratios, i.e. when either the numerator or denominator can be reduced to 1, the rhythm is easier to learn than when this is not the case [14], [15]. Tapping the left index finger twice as fast as the right one, 2∶1, is easier than tapping it 1.5 times as fast, as in 3∶2 [16], [17].

Relatively few systematic studies have compared the acquisition of increasing frequency ratios with tasks other than finger tapping. Some studies directly compared tapping with continuous drawing and found a low correlation between these two types of movement [18]. Acquisition of a novel task is highly dependent on movement topology, as such, the extent to which coordination constraints can be applied across categories of movement is not a trivial question [19], [20].

In addition to temporal complexity, bimanual coordination is also determined by handedness, directionality, and the muscle grouping constraint [6], [21], [22], [23], [24], [25], [26], [27]. With respect to direction, isodirectionality in extrinsic space generally results in better performance than non-isodirectionality, though this is highly dependent on the limbs involved as well as the rate at which they move [11], [28]. Regarding handedness, if the non-dominant hand is required to move at a faster speed, then performance is usually worse than during the converse arrangement [29], [30]. Most studies that have examined the role of hand dominance included asymmetrical movements where the left and right hand each performs a unique trajectory [31]. In such studies, the dominant hand influences performance such that dimensions of the shape created by the non-dominant hand begins to contain features of the shape created by the dominant hand [20], [29], [32], [33], [34]. Moreover, muscle grouping refers to the body's natural preference to co-activate homologous muscles, i.e. simultaneous contraction of a pair of flexors alternated with extensors is more stable than the simultaneous contraction of a flexor and an extensor [2], [6], [27], [35] for exceptions see [27], [36]. The former is the more favorable state and is referred to as ‘in-phase’, whereas the latter is slightly less stable and referred to as ‘anti-phase’ [1]. With some exceptions, coordination constraints have often been addressed in separate experiments. To our knowledge, this is the first study that utilizes a single task to address all of the aforementioned coordination constraints together. In so doing, we are in a position to quantify the strength and interactions among constraints.

Independently, these various constraints have been demonstrated to be quite powerful [26], [37]. As such, it is important to determine the extent to which these robust coordination constraints may be overcome with practice. Practice in the presence of visual feedback is a key component of learning [38], [39]. The type of visual feedback most widely used in cyclical bimanual tasks occurs during the trial in the form of Lissajous plots, named for the French mathematician [40], [41], [42], [43], [44], [45], [46]. Lissajous plots are graphs of parametric equations whose shape is highly sensitive to the ratio a/b. Notably, the plots vary dramatically in perceptual complexity across various frequency and directional combinations [28]. For example, if the left and right hand move at the same frequency the result may be either a line, a circle, or an ellipse, depending on the relative phasing. If the frequency ratio changes to 2∶1, the dimensions of the visual stimulus become more complex, i.e. the letter C or the number eight where the configurations depend on relative phasing. Therefore, while they are useful for guiding performance, the large variation in visual cues can make direct comparison across various trial types difficult. The increasing evidence for the tight interactions between perception and motor learning increases the demand for a task where visual cue complexity can remain constant across different trial types [36], [47], [48], [49], [50]. Here we resolve each of the above issues using a simple perceptual cue that remains relatively constant across all frequency and directional combinations, and by systematically testing isofrequency and multifrequency patterns with a task that requires constant motion.

There were two main objectives of this experiment: (1) to test the interaction of multiple coordination constraints with a single task, (2) to determine the extent to which constraints would be overcome with practice. Several task variants were practiced in parallel. The task resembles the popular “Etch-a-Sketch” toy (for early versions of this task, [51], [52]). Here, continuous cyclical rotational movements were required by both hands. Augmented visual feedback was provided on a PC screen which integrates the produced hand movements into a unified visual display. In this new paradigm, we used a simple perceptual cue, a straight line, which remains constant in length across all frequency and directional combinations. Only the angle of the line changed, which depended upon the frequency and rotational direction of the dials. To analyze bimanual coordination, we used dependent measures that could be easily applied across all variations of the task – regardless of the direction, temporal requirements, or hand dominance. Based on the previous literature, we predicted each one of the following: (1) isofrequency patterns would result in less error than multifrequency patterns, (2) frequency ratios with integers would result in less error than those with non-integers, (3) conditions in which the dominant hand was required to move faster would result in less error than instances in which the non-dominant hand was required to do so, (4) in-phase would result in less error than anti-phase, and (5) practice conditions would result in overcoming each of these bimanual constraints.

## Methods

### Participants

Twenty-two healthy young adults (6 male, 16 female; mean age = 23.6 years, SD = 2.3, range 20–27) without known muscular disorders participated in this experiment. All subjects were right handed, as determined by an adapted version of the Edinburgh Handedness Inventory (mean laterality = 87.6, SD = 16.5, range 53–100) [53]. They were naïve with respect to the task and had normal or corrected-to-normal vision.

### Ethics Statement

Informed consent was signed by every participant prior to testing. The experiment was approved by the local Ethics Committee of K.U.Leuven and was performed in accordance with the 1964 Declaration of Helsinki.

### Apparatus and task description

**Figure 1:**
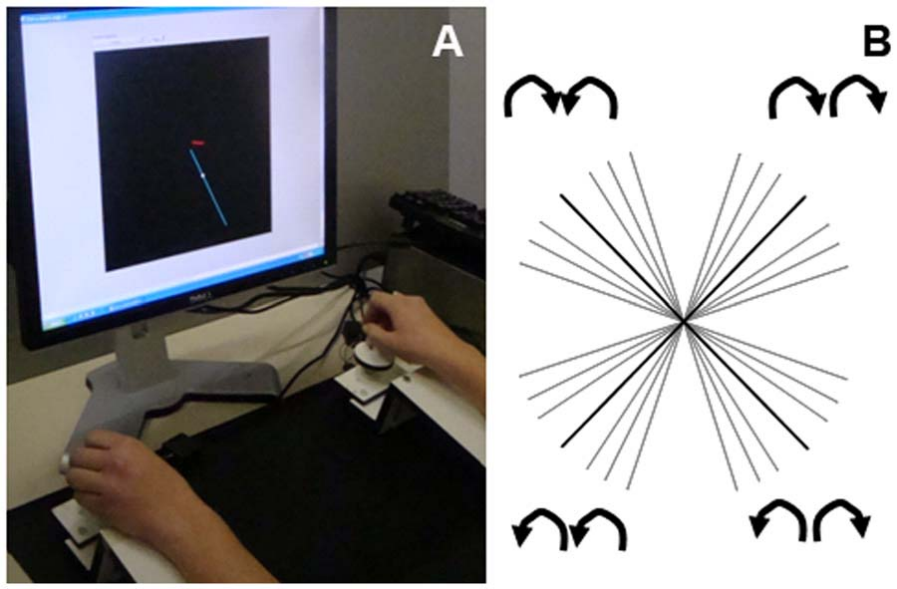
A. A schematic of 28 possible lines representing different bimanual coordination patterns. When lines occur closer to the vertical (y) axis, the left hand is rotating faster. When lines occur closer to the horizontal (x) axis, the right hand is leading. A bold line that is a 45° angle indicates that the left and right hands are rotating at an equal rate (isofrequency). B. View of the experimental apparatus (please note that the hands are normally covered to prevent their vision).

Participants were comfortably seated at a table in front of a computer monitor with both lower arms resting on two custom-made adjustable ramps. At the end of each ramp, 8 cm below the plane of the ramp, a dial was mounted on a horizontal support consisting of a flat disc (diameter 5 cm) and a vertical peg. The dials were rotated by holding each peg between the thumb and index finger, i.e. similar to the position assumed when holding a pencil. High precision shaft encoders were aligned with the axis of rotation of the dials to record angular displacement (Avago Technologies, 4096 pulses per revolution; accuracy = .089°, sampled at 100 Hz). The wrists rested at the edge of the ramp covered with foam to maximize comfort and minimize fatigue. Direct vision of both hands and forearms was occluded by a horizontal table-top bench that was placed over the forearms of the subject (Figure 1).

**Figure 2:**
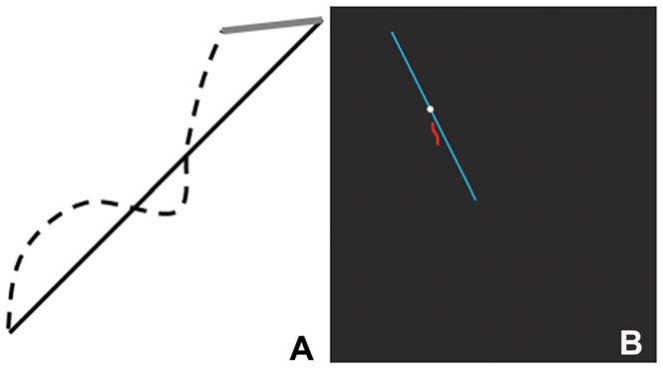
A schematic of the two dependent measures of performance. Solid line represents the target line that the subjects must trace. Dashed line represents a hypothetical path of the subject. Finish offset error (FO) is the hypotenuse of the right triangle that is formed from the end of the subject's path and the end of the target line. Absolute deviation (AbDv) is the area between the subject's path and the target line.

The two dials controlled movement of a red cursor (a flexible line segment approximately 1 cm long) on the computer monitor (Figure 2). The left and right dial controlled this red cursor's movement along the vertical and horizontal axis, respectively. When the left hand dial was rotated to the right (clockwise), the cursor moved up; when turned to the left (counterclockwise), the cursor moved down. When the right hand dial was rotated to the right (clockwise), the red cursor on the screen moved to the right, when rotated to the left (counterclockwise), the cursor moved to the left. The target was a white cursor moving from the center of the display (a black square 15×15 cm), along a blue target line, to the periphery, indicative of the bimanual coordination pattern to be produced. The gain was set to 10 arbitrary units per rotation so that, to complete a horizontal or vertical line approximately 15 cm long, one dial was rotated 15 complete cycles. The gain and the time to complete a line (7 sec) were selected based on pilot data. With the exception of the isofrequency pattern, the cycles required by the left and right hands varied depending on the frequency ratio. For isofrequency patterns, the left and right hands moved at 1.7 Hz per line.

The monitor was approximately 51 cm along the diagonal, and the target diagonal line on the screen was approximately 10 cm in length. Computer programming for this task was done using LabView, version 8.5. When both dials were turned simultaneously, the cursor moved at an angle that was dependent on both the *direction* and *frequency* of dial rotation. Regarding direction, this gave rise to four distinct bimanual movement patterns: rightward (clockwise), leftward (counterclockwise), inward, outward. Whereas the combined *direction* of the dials determined in which quadrant the cursor would travel, the combined relative *frequency (frequency ratio)* determined the precise angle of the line (slope). For example, if the left and right hand dials moved at the same rate in a clockwise direction, then a line segment with a 45° angle would be produced; if the left hand moved twice as fast as the right hand (frequency ratio of 2∶1), the angle became 63°. When describing the unique Frequency Ratio, we adopted the convention of always referring to the left hand first, and the right hand second, LH∶RH. Notably, when both hands were rotating clockwise or counterclockwise at the same cycling frequency (1∶1 only) movements were associated with the anti-phase coordination mode; when hands were rotating inward or outward at the same cycling frequency (1∶1 only) movements were associated with the in-phase coordination mode. When the hands moved at relative angular velocities not equal to 1, then this phase notation was no longer applicable. The relative angular velocity simply refers to the value of the frequency ratio without respect to which hand is rotating faster. For 2∶1 and 1∶2, the relative angular velocity was 2; for 3∶1 and 1∶3 the relative angular velocity was 3; and for 3∶2 and 2∶3, the relative angular velocity was 1.5. The experimental conditions were counterbalanced for the hand that was assigned with the faster cycling frequency (either the dominant or nondominant hand). We tested seven frequency ratios: 3∶1, 2∶1, 3∶2, 1∶1, 2∶3, 1∶2, 1∶3. The combination of movement directions (4) and frequency ratios (7) resulted in 28 experimental target pathways (See Figure 1, left).

A trial included presentation of a single target line with a distinct angle representing a unique coordination pattern. The starting point and total length of the target line remained constant across task conditions. Once the target line was displayed, a target cursor (white moving dot) remained motionless at the origin for 200 ms, after which it began to move along the line at a constant rate and for a total duration of 7 seconds for each 10 cm line. Participants were instructed to track the target as accurately as possible. After 7 seconds, the line disappeared and the display returned to black. The trial ended regardless of the subject's location on the screen. The time between trials (intertrial interval) varied randomly between 4 and 6 seconds. Each trial required a unique bimanual coordination pattern – proper direction and angular velocity of both dials – to produce a line at the correct angle. In other words, subjects had to match the red cursor with the white target in both space and time.

Participants were randomly assigned to two different groups: Practice (n = 9) and No Practice (n = 13). Both groups were tested on two occasions, i.e. pretest and a posttest, which occurred one week after pre-test or the last practice session. One trial block included 28 randomized trials, i.e. 4 possible directions and 7 Frequency Ratios so that each coordination pattern was tested once. This was repeated six times with 1–3 minutes of rest between each block of trials. Participants of the Practice Group practiced the experimental tasks for four additional days in between pre- and posttest. The four training days were identical to the pre- and post-tests. Prior to data recording, participants were given 4 practice trials to become familiar with the task. The training session typically lasted 40 minutes.

### Dependent measures

The data of the bimanual coordination task was analyzed using Labview (8.5) software (National Instruments, Austin, Texas, USA) and Matlab R2008a. On each trial, the x- and y- positions of the target and the cursor were sampled in real time at 100 Hz. Subsequent off-line processing was carried out using Matlab R2008a and Microsoft Excel 2007. Measures of accuracy consisted of two dependent variables which were calculated per target line: finish offset error (finish offset error) and absolute deviation (AbDv) (Figure 2). Finish offset error indicated the difference between the target position and the cursor position at the end of each trial, calculated using the Euclidean distance:
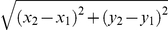
Where 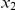 and 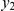 refer to the endpoint of the subject's line on the x- and y-axis, respectively and 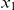 and 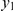 correspond to the endpoints of the target line on the x- and y- axis, respectively. A finish offset error that is equal to 0 indicates that the red cursor was precisely on top of the white target at the end of the trial, representing a perfect performance. Accordingly, the larger the finish offset error is, the poorer the performance. AbDv was calculated based on the amount of divergence from the target line expressed as area under the curve. Deviation from the target line was sampled at a rate of 100 Hz and summed, excluding time points in which the cursor remained motionless. All dependent variables were transformed into z-scores [(X – MEAN)/SD)]. A trial was classified as an outlier and discarded from the analysis when z values were greater than |3|. On average 3% of the data points were removed from the dataset. The main advantage of both measures is that they were applicable to the various coordination tasks, irrespective of their frequency ratio's.

### Statistical Analysis

To test the role of frequency ratio, relative angular velocity, hand allocation and directionality in this novel visuomotor task, we performed several ANOVAs using both dependent measures. First, outcome measures of the bimanual coordination task (finish offset error and AbDv) were analyzed using 2×2×4×7 (Group×Day×Direction×Frequency Ratio) ANOVA. Levels for each factor were as follows: Group (Practice and No Practice), Day (Pre- and Post-test), Direction (Leftward, Rightward, Inward, and Outward) and Frequency Ratio (3∶1, 2∶1, 3∶2, 1∶1, 2∶3, 1∶2, 1∶3). Based on this, Direction was collapsed from 4 levels to 2 levels such that Leftward and Rightward were combined and Inward and Outward were combined. Hence, the reduced ANOVA used for further analysis was 2×2×2×7 (Group×Day×Direction×Frequency Ratio). Second, we compared isofrequency patterns using a 2×2×2 (Group×Day×Direction) repeated measures ANOVA to investigate the effect of inphase/antiphase coordination patterns. Leftward and rightward were collapsed and inward and outward were collapsed. Third, to determine the effect of relative angular velocity and faster hand, a 2×2×3 (Group×Fast Hand×Relative Angular Velocity) ANOVA with repeated measures was performed. Finally, we determined changes in motor performance for the Practice Group only across the four practice days using a 6×2×7 (Day×Direction×Frequency Ratio) ANOVA with repeated measures. The associated p-values for each F-statistic were adjusted via Greenhouse-Geisser for violation of sphericity assumption. Significant main and interaction effects were further explored by post hoc tests using Bonferroni correction. All statistical analyses were performed with Statistica 8 (StatSoft, Inc. Tulsa, OK) using an α-level of 0.05.

## Results

### Finish offset error

**Figure 3:**
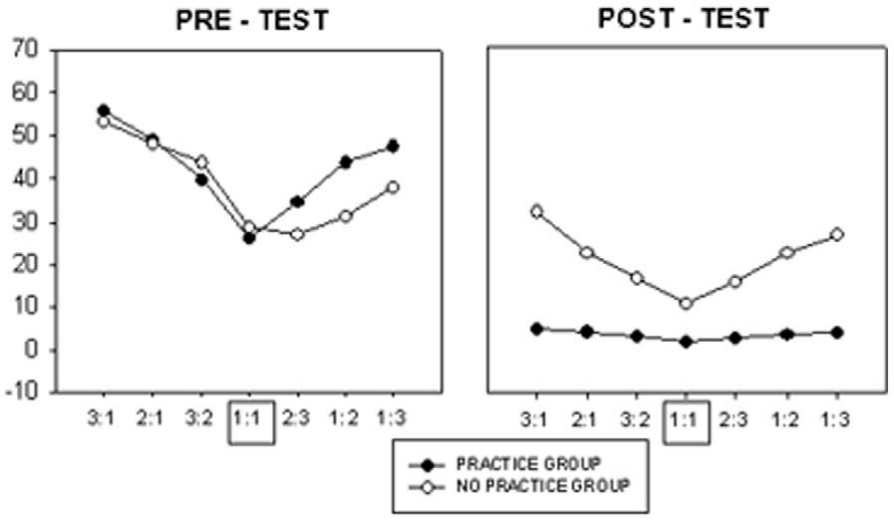
Three-factor interaction of finish offset error for Day, Group, and Frequency Ratio. The 1∶1 pattern resulted in the smallest error and the non 1∶1 ratios resulted in greater error. The pattern has the shape of a ‘seagull’. Frequency Ratios where the left hand rotates faster are represented by the left wing and Frequency Ratios where the right hand moves faster are represented by the right wing. After 4 days of practice, all coordination constraints are overcome, as indicated by the straight line.

We hypothesized that finish offset error would be greatest for the largest Frequency Ratios, e.g. 3∶1 and 1∶3, and would decrease as the Frequency Ratios approached 1∶1, with possible directional interactions. We also predicted that, at Post-Test, finish offset error would be smaller for the ‘Practice’ group compared with the ‘No Practice’ group. To test this, finish offset error was studied using a 2×2×2×7 (Group×Day×Direction×Frequency Ratio) ANOVA with repeated measures. The Day, Direction, and Frequency Ratio main effects were significant (see Table 1). Finish offset error decreased from day of Pre-Test (40±3) to day of Post-Test (12±1) indicating that the subjects successfully learned the task (p<.05). Finish offset error for the leftward-rightward direction (24±2) was smaller than for that of the inward-outward direction (28±2) (p<.05). Regarding Frequency Ratio, the 1∶1 pattern resulted in the smallest finish offset error (17±1) followed by 2∶3 (20±2). The greatest error occurred when the Relative Angular Velocity between the two hands was the greatest, and the left hand was required to rotate faster, 3∶1 (36±3). A significant Group×Day interaction was observed indicating that finish offset error decreased to a larger extent between pre- and post-test in the group that practiced compared with the control group. In addition, a significant interaction was detected for Day×Frequency Ratio. The overarching Group×Day×Frequency Ratio interaction was also significant (Figure 3). As expected, the modulation of performance as a function of Frequency Ratio was similar for both groups on day of pre-test, where a clearly ordered pattern was observed (Figure 3, left side). Finish offset error was smallest for the 1∶1 Frequency Ratio. As the frequency difference between the two hands gradually increased, the error increased as well, resulting in a seagull pattern. There was a slight asymmetry to the pattern (i.e., a higher left than right wing) – ratios requiring the non-dominant left hand to move faster tended to result in higher finish offset error than the ratios that required the dominant right hand to move faster. Conversely, at posttest, the No-Practice Group still exhibited the seagull pattern whereas performance error across the different Frequency Ratios became very similar in the Practice Group, resulting in a flat line (Figure 3, right side).

**Table 1:** Summary of ANOVA results on Finish Offset error and Absolute Deviation (2×2×2×7).

	*Finish Offset Error*			*Absolute Deviation*		
	*df*	*F*	*P*	*df*	*F*	*P*
Group (Grp)	1,20	1.84	0.190	1,20	4.77	**0.041**
Day	1,20	76.53	**0.000**	1,20	130.30	**0.000**
Direction (Dir)	1,20	5.95	**0.024**	1,20	8.17	**0.009**
Frequency Ratio (FR)	6,120	38.04	**0.000**	6,120	8.55	**0.001**
Grp×Day	1,20	10.71	**0.003**	1,20	13.43	**0.001**
Grp×Dir	1,20	1.58	0.222	1,20	0.71	0.406
Grp×FR	6,120	2.47	0.072	6,120	1.26	0.288
Day×Dir	1,20	1.08	0.309	1,20	2.66	0.118
Day×FR	6,120	9.31	**0.000**	6,120	1.22	0.307
Dir×FR	6,120	8.93	**0.000**	6,120	1.35	0.258
Grp×Day×Dir	1,20	0.64	0.433	1,20	1.05	0.315
Grp×Day×FR	6,120	6.77	**0.001**	6,120	1.99	0.129
Day×Dir×FR	6,120	1.96	0.120	6,120	1.17	0.327
Grp×Dir×FR	6,120	5.74	**0.000**	6,120	0.78	0.526
Grp×Day×Dir×FR	6,120	0.78	0.520	6,120	1.21	0.311

**Figure 4:**
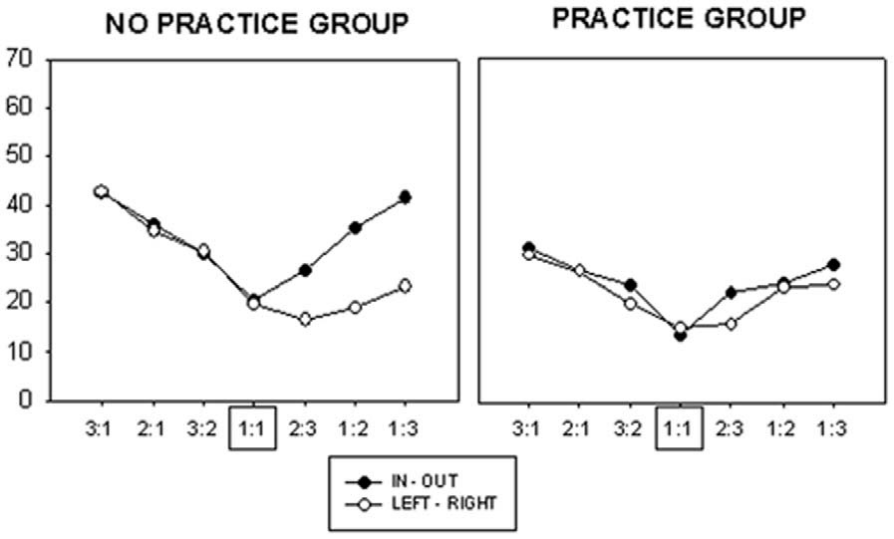
Three-factor interaction of finish offset error for Group, Direction, and Frequency Ratio. The ‘seagull’ pattern is clear when the direction required is inward or outward. However, when the required direction is left or right, an asymmetrical pattern emerges. When the dominant right hand was required to rotate faster, the finish offset error was smaller than when the non-dominant left hand was required to lead.

The Direction×Frequency Ratio interaction as well as a Group×Direction×Frequency Ratio interaction also reached significance (Table 1). In the ‘No Practice’ group, finish offset error was smaller for the leftward-rightward direction than for the inward-outward direction, but only for Frequency Ratios where the dominant hand moved faster (Figure 4). No such effect was observed in the group that practiced.

#### Isofrequency (1∶1)

We hypothesized that in-phase would lead to smaller finish offset error than anti-phase. To evaluate the in-phase versus anti-phase constraint, a 2×2×2 (Group×Day×Direction) ANOVA with repeated measures was computed for the 1∶1 conditions only. There was only a significant main effect of day [F(1,20) = 48.11, p<0.01]. Finish offset error decreased from 27±3 to 6±0.8 between day of pre- and post-test (p<.05). No main effect of Direction was found.

#### Hand allocation and relative angular velocity between hands

We hypothesized that when the left hand was required to rotate faster, finish offset error would be greater than when the right hand was required to do so. A 2×2×3 (Group×Faster Hand×Relative Angular Velocity) ANOVA with repeated measures revealed a main effect of Day, Faster Hand, and Relative Angular Velocity (Table 2). Overall, finish offset error decreased between day of pre- and post-test from 42±4 to 13±2 (p<.05). It was smaller when the dominant right hand was rotating faster, 24±2, compared with the non-dominant left hand, 31±2 (p<.05). In addition, finish offset error was smallest when the Relative Angular Velocity between the two hands was the smallest, i.e. 1.5 (23±2) and it was largest when Relative Angular Velocity between the hands was also the largest, i.e. 3.0 (32±3) (p<.05). The mean finish offset error for Relative Angular Velocity of 2.0 was 28±2. A significant Relative Velocity×Fast Hand interaction was obtained, indicating that finish offset error depended on which hand was required to move more quickly. When the non-dominant left hand was required to move more quickly, finish offset error was greater than when the dominant right hand was the faster hand.

**Table 2:** Summary of ANOVA results on Finish Offset Error and Absolute Deviation (2×2×3).

	*Finish Offset Error*			*Absolute Deviation*		
	*df*	*F*	*P*	*df*	*F*	*P*
Group (Grp)	1,20	1.73	0.203	1,20	4.36	0.050
Faster Hand (FstH)	1,20	23.62	**0.000**	1,20	0.052	0.821
Relative Angular Velocity (RV)	2,40	54.93	**0.000**	2,40	11.92	**0.001**
Grp×FstH	1,20	4.76	**0.041**	1,20	0.807	0.379
Grp×RV	2,40	1.44	0.249	2,40	1.89	0.182
RV×FstH	2,40	4.34	**0.021**	2,40	3.56	**0.038**
Grp×FstH×RV	2, 40	0.65	0.518	2, 40	0.80	0.450

#### Practice Group across four days of training

**Figure 5:**
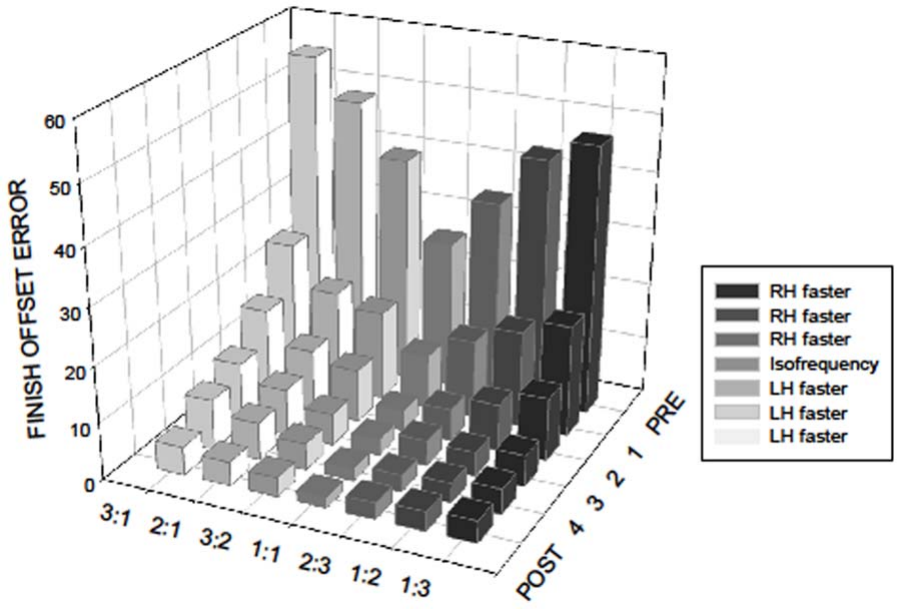
Four days of practice with augmented visual feedback overcomes all of the coordination constraints tested.

We hypothesized that finish offset error would decrease with practice. Change in finish offset error across days for the Practice Group only was determined using a 6×2×7 (Day×Direction×Frequency Ratio) ANOVA. There was no significant main effect of direction, but there was a significant main effect of Day [F(5,40) = 26.83, *p*<.01] and Frequency Ratio [F(6,48) = 9.81, *p*<.01]. There was a significant interaction effect between Day and Frequency Ratio [F(30,240) = 3.85, *p*<.01], which demonstrated that the biggest improvement in finish offset error occurred in the condition that had the greatest error initially (Figure 5, far left and far right Frequency Ratios). Bonferroni post hoc tests revealed that there was a significant decrease between pre-test and day 1 of practice (p<.01) as well as between day 1 and day 2 of practice (p<.01). No significant differences in performance were observed between days 3 and 4 and day of post-test (p<.01). That is, by day 3, performance reached asymptote levels. Furthermore, there were no significant differences in performance between Frequency Ratios at post-test (p>.01).

### Absolute Deviation

**Figure 6:**
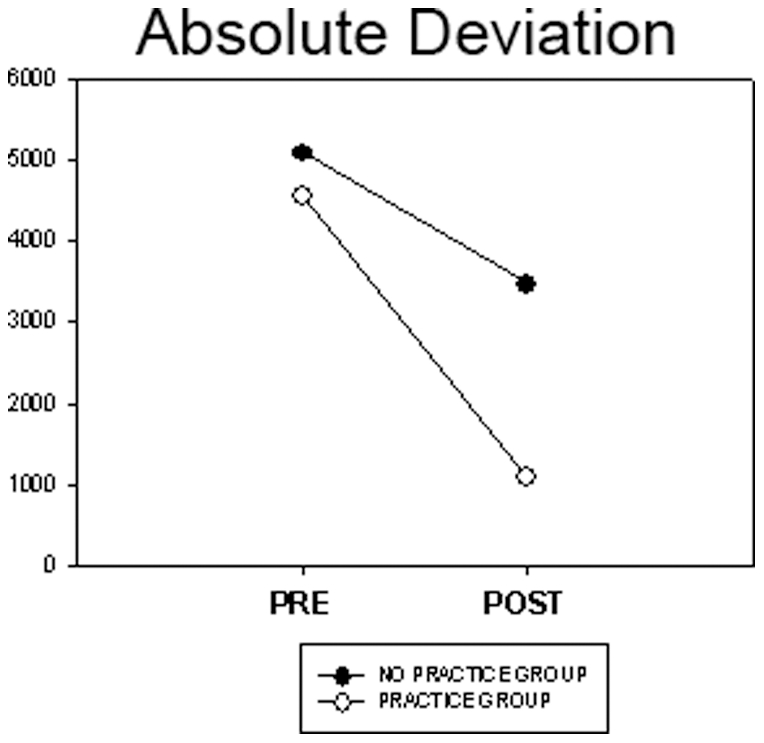
Two-factor interaction of absolute deviation for Day and Group. On day of post-test, the group with four days of practice performed significantly better than the group with no practice.

We hypothesized that changes in AbDv would be similar to the pattern observed with finish offset error. That is, AbDv would be greatest for the largest Frequency Ratios and would decrease as the Frequency Ratios approached 1∶1, with possible directional interactions. We also predicted that AbDv would be smaller for the ‘Practice’ group compared with the ‘No Practice’ group at Post-Test. To test this, AbDv was compared using a 2×2×2×7 (Group×Day×Direction×Frequency Ratio) ANOVA with repeated measures. We observed a significant main effect of Group, Day, Direction, and Frequency ratio (Table 1). The Practice Group had a smaller AbDv compared with the No Practice Group (2841.01±511.17 and 4294.04±425.32, respectively) (p<.05). AbDv decreased between day of pre- and post-test (4840±403.58 and 2294.43, respectively) (p<.05). The leftward-rightward direction resulted in a smaller AbDv than the inward-outward direction (3428.20±350.12 and 3706.84±321.34) (p<.05). The Day×Group interaction was significant (Figure 6). Whereas performance levels for both groups were similar at pretest, error scores decreased more sharply at post-test for the Practice Group as compared to the No-Practice Group. The remaining interactions were not significant.

#### Isofrequency

We hypothesized that in-phase would result in smaller AbDv than anti-phase, and that the ‘Practice’ group would perform better than the ‘No Practice’ group on Day of Post-Test. A 2×2×2 (Group×Day×Direction) ANOVA with repeated measures was calculated. There was a main effect of Group [F(1,20) = 6.81, *p*<.01] and Day [F(1,20) = 53.20, *p*<.01]. AbDv was smaller in the Practice Group (2057.34±249.07) compared with the No-Practice Group (2903.38±207.24) (p<.05) and it showed a tendency to decrease between day of pre- and post-test which was marginally significant (3575.16±297.21 to 1385.56±95.98) (p = .09).

#### Hand allocation and relative angular velocity between hands

We hypothesized that as the Relative Angular Velocity between the hands increased, the AbDv would increase. We also predicted that AbDv would be greater when the Fast Hand was the non-dominant, left hand. A 2×2×3 (Group×Fast Hand×Relative Angular Velocity) ANOVA with repeated measures revealed a significant main effect of Group, and Relative Angular Velocity, but not Fast Hand (Table 2). The Practice Group had smaller AbDv than the No Practice Group (p<.05). In addition, as the Relative Angular Velocity between the hands increased, AbDv increased as well (p<.01). The only significant interaction was between Fast Hand and Relative Angular Velocity: when the non-dominant left hand was required to move more quickly, AbDv was greater than when the dominant right hand was the faster hand.

#### Practice Group across four days of training

We predicted that AbDv would decrease with practice with possible directional interactions. Consistent with finish offset error, we predicted that AbDv would decrease as the Frequency Ratios approached 1∶1, regardless of integer or non-integer ratio. Change in performance across days for the Practice Group only was further evaluated using a 6×2×7 (Day×Direction×Frequency Ratio) ANOVA with repeated measures. All main effects were significant. AbDv decreased across days [F(5,40) = 69.73, *p*<.01]. Similar to finish offset error, 1∶1 resulted in the smallest error and 3∶1 resulted in the greatest error at pre-test (p<0.05). However, after four days of training, AbDv was very small and did not differ across any of the Frequency Ratios tested (p>.05).

## Discussion

The purpose of this study was two-fold. First, a novel task was designed to test the interaction of multiple bimanual coordination constraints. Second, we determined the extent to which these constraints may be overcome with practice in the presence of augmented feedback. The most novel finding of this report occurred in the pattern of multifrequency ratios. Contrary to the finger-tapping literature, we discovered that the non-integer ratios (3∶2 and 2∶3) resulted in better performance than the integer ratios, (3∶1, 1∶3, 2∶1, and 1∶2). This was true regardless of which hand was required to move at a faster rate. We found that as the difference in angular velocity between the two hands increased, accuracy decreased. Hand dominance was an important factor; error was greater when the non-dominant left hand was required to rotate faster compared with the right hand. In addition, four days of practice led to significant reductions in error across all frequency and directional combinations. At time of post-test, performance was comparable across all Frequency Ratios, but only in the ‘Practice Group’, demonstrating that with proper training and augmented visual feedback, multiple coordination constraints can be overcome. These findings are discussed in detail next.

### Isofrequency and multifrequency rhythms

In this task, only the 1∶1 trials corresponded with in-phase and anti-phase patterns. These types of isochronous movements have been investigated extensively using the Haken-Kelso-Bunz (HKB) model [54]. In the HKB model, bimanual coordination is modeled according to coupled-oscillators where in-phase and anti-phase represent two attractor states, or local minima. Notably, the former is slightly stronger than the latter. The model predicts that as speed increases, the tendency to transition from anti-phase to in-phase is amplified. In the present study, no significant differences were found between in-phase and anti-phase coordination patterns which may be partly due to the relatively low speed requirements in addition to task differences. Although constraints were not evident in this 1∶1 mode, we do not interpret this to mean that they do not exist. The muscle grouping constraint may be overcome when visual cues that guide movement are represented in a simple and unified manner [36], [55]. Similarly, an increasing number of studies are demonstrating its dependency on both movement topology and perceptual cues [2], [27], [36]. In addition, studies that examine in-phase and anti-phase kinematics are often restricted to performance of isofrequency rhythms only. In the present study, in-phase and anti-phase represented only a subset of the task variants – 2 of the 28 coordination patterns were in-phase and 2 were anti-phase. As such, contextual embedding or interference effects may have masked the in-phase vs. anti-phase effect.

The muscle grouping constraint has been demonstrated to exhibit a significant interaction with directionality [22], [56]. Thus, the trajectory of the movement is indeed an important factor in understanding coordination constraints. Our finding that the isochronous pattern resulted in the smallest error is consistent with previous research making use of various movement types [57], [58], [59]. For example, when 1∶1, 2∶1 and 3∶1 were compared using elbow movements, variability increased as relative velocity between the limbs increased [30]. With finger oscillations as well, isofrequency ratios are generally found to be more stable than multifrequency ratios [9]. Thus, the innate preference for moving the upper limbs at the same rate is a highly robust coordination constraint observed across different limbs and types of movement.

One primary objective of this experiment was to directly compare a variety of multifrequency patterns with a task that was distinctly different from the finger tapping paradigm. The most striking feature to emerge from our analysis was the order that we observed across Frequency Ratios (Figures 3 and 4). The isofrequency ratio (1∶1) resulted in the smallest error scores and it increased as the difference in angular velocity between the hands increased. With Frequency Ratio plotted on the x-axis (1∶1 at the center) and error on the y-axis, the pattern resembled the shape of a ‘seagull.’ The left wing represents ratios where the left hand rotates faster whereas the right wing represents ratios where the right hand moves faster (Figures 3 and 4). Interestingly, this sequence was followed regardless of whether or not it was an integer ratio. As already mentioned, previous literature has shown that integer ratios (greater than one) tend to lead to lower error than non-integer ratios [7], [15], [16]. Contrary to these findings, we observed that the integer ratios resulted in greater error than non-integer ratios - with the single exception of the intrinsically favorable isofrequency pattern. The 3∶1 combination was the most difficult, followed by 2∶1, then 3∶2, and finally, 1∶1. The most likely explanation for this result is the relative velocity between the two hands. When performing the 3∶2 ratio, the relative angular velocity between the two hands is 1.5, whereas, it is 2 and 3 for 2∶1 and 3∶1 combinations, respectively. The 1∶1 mode is a highly stable, attractor state [6]. Hence, to perform any of the multifrequency rhythms, the natural tendency to move isochronously must be inhibited or suppressed. In essence, the hands must ‘decouple’. As the Frequency Ratio moves progressively farther from 1, the degree of decoupling that is required also progressively increases. These data suggest that the small error of the 3∶2 pattern is perhaps due to its proximity to the isofrequency state and the incrementally larger finish offsets of the 2∶1 and 3∶1 patterns are due to their increasing distance from the 1∶1 state [60].

The divergent findings from the finger tapping work may be due to the different kinematics of this dial rotation task. Although periodicity was not directly tested here, the hand movements can be regarded as much more continuous and smooth than finger tapping movements that require distinct reversals in direction. Correspondingly, the disagreement with the previous literature on integer and non-integer ratios can possibly be accounted for by these different movement types and their associated timing, i.e. discrete events such as finger tapping (typically used in past polyrhythm research) versus emergent timing (as used in the present bimanual circling task) [61]. Moreover, consistent with the more discrete finger tapping motion, the sensory cue used for finger tapping is a discrete auditory metronome. The sensory modality and the stimulus properties of the feedback are also important factors for bimanual learning [50]. The role of augmented visual feedback is discussed in more detail below.

### Hand allocation and directionality

We also considered the effects of both hand allocation and directionality on the production of multifrequency patterns. Main effects with respect to faster hand, directionality, and test day were significant, as well as the 3-factor interaction of faster hand, directionality and practice (Table 2, Figure 4). In the ‘No-Practice’ Group, if one looks only at the ‘left wing’ of the seagull, 3∶1, 2∶1 and 3∶2, it is clear that error is similar for both directions (leftward/rightward vs. inward/outward). If one now looks at the ‘right-wing’ within this same graph, 1∶3, 1∶2, 2∶3, the error scores are dissimilar across both directions. Within this subset, finish offset error is greater when the required rotations are inward and outward compared with trials where the required rotations are leftward and rightward.

It is well established with isofrequency rhythms that inward and outward directions, which represent mirror-symmetric movements, are a highly stable, and intrinsically favorable state [6], [21], [23], [41]. While the present conditions represent multifrequency patterns, it is possible that error is smaller in the leftward-rightward directions, because in this case, the constraint that must be overcome is not as strong as that of inward-outward rotations, i.e. a weaker ‘magnet effect’ [5]. With respect to the Practice Group, the difference between directions for the ratios where the right hand moves faster was not observed. This seems largely due to the practice-induced reduction in error which occurred for the inward-outward direction with respect to Frequency Ratios, 1∶3, 1∶2, and 2∶3.

We did not include left handed subjects in this experiment, however, within right handed subjects, the present study appears to be consistent with previous work which demonstrates that hand dominance influences bimanual coordination [62], [63], [64]. Performance was generally worse when the non-dominant hand was required to rotate faster as compared to the dominant hand for any given Frequency Ratio. As previously mentioned, hand dominance interacted with directionality. This effect was most remarkable when rotations were leftward/rightward in the ‘No Practice’ Group (Figure 4, left). In the ‘Practice’ Group, the symmetry of the ‘seagull’ pattern was evident (Figure 4, right). This demonstrates that the hand dominance constraint was overcome after 4 days of training with augmented visual feedback.

### Overcoming bimanual constraints with practice

The results demonstrated conclusively that the training provided to the Practice Group – four days with augmented visual feedback—was adequate for overcoming coordination constraints. At pre-test, performance was similar between the ‘Practice’ and ‘No Practice’ groups. At post-test, the ‘seagull’ effect was only evident in the ‘No Practice’ Group. In the ‘Practice’ Group, it became essentially a flat line (Figure 3). The subjects gained expertise on this task as indicated by their considerable reduction of error. The learning approach in the present study differs from previous work in that a broad range of coordination patterns was acquired simultaneously. Specifically, subjects learned multiple coordination patterns requiring various temporal and directional combinations – 28 in all.

A primary aim of this experiment was to measure the extent to which multiple coordination constraints would be overcome after several days of practice. The task was explicitly designed to be challenging for subjects and task variation likely induced contextual embedding or interference effects [65]. That is, the speed and directional combinations varied from trial to trial and participants could not anticipate or plan movement patterns until the stimulus (target line) was displayed on the screen.

An increasing number of studies have demonstrated the important role of perception in guiding bimanual coordination [2], [40] [48], [56] [66], [67]. In the present task, the properties of the visual feedback differed from previous work in our laboratory in that a single line was used for representing coordination patterns visually instead of the Lissajous plots. The Lissajous plots increase in perceptual complexity as the Frequency Ratios change. For example, when the left and right hand move at an isofrequency rhythm, 1∶1, the result may be either a line, a circle, or an ellipse. The Lissajous plot is dependent on the relative phasing. Visual complexity further increases if the left and right hands move at different rates. While such plots are useful for guiding performance, the large variation in visual cues can make direct comparison across various trial types difficult. Although Lissajous plots differ from the visual stimuli used in the present study, both feedback types are similar in that they integrate movement into a unified visual display, concurrent with ongoing performance. Importantly, the increasing evidence for the tight interactions between perception and motor learning increases the demand for a task where the relevant perceptual cues can be controlled across different trial types. Here we used a straight line for all frequency and directional combinations, while only the angle (slope) of the line changed. In so doing, the shape and dimensions of the perceptual cue was preserved across trial types and differed only with respect to its position in allocentric space. It is possible that the constancy and simplicity of the cue in this task may have facilitated the subject's ability to overcome constraints. Indeed, the ability to easily interpret the visual stimulus is an important factor in overcoming coordination constraints [2], [20], [36], [40], [48], [55].

Moreover, after four days of practice, error was extremely small, similar to that observed with the intrinsically favorable 1∶1 pattern. However, it is important to note that this successful performance was obtained in the presence of augmented feedback at all times. It is well known that subjects can become overly dependent on the augmented feedback and may fail to demonstrate strong retention when augmented feedback is removed [39], [56], [68], [69]. The type of augmented visual feedback used in this study has been demonstrated to facilitate acquisition of a new task, however it can actually impede consolidation or retention when compared to other modalities, such as auditory feedback [50]. This may be due to the fact that augmented visual information may become part of the movement representation such that the brain areas involved in processing of this augmented feedback are still activated when that feedback is removed [50], [70]. This suggests that generalization of performance under nonaugmented feedback conditions remains to be investigated as well as the conditions in which such powerful feedback can be maximally exploited to benefit the learner while minimizing dependence on this source of information.

In this study, we examined each of the following bimanual constraints: in-phase versus anti-phase, hand allocation, directionality and isofrequency versus multifrequency. By incorporating each of these into a single task, we were able to examine the extent to which these constraints interact with one another. Recent work has demonstrated that bimanual coordination constraints may not follow a strict hierarchy, but rather may change depending on the task demands [71]. Consistent with this, we found that the in-phase versus anti-phase constraint was not dominant in this bimanual tracking task. Rather, the Frequency Ratio seemed to be the most important factor in determining error: As the Frequency Ratio grew increasingly farther away from 1, error increased, regardless of whether the ratio was integer or non-integer. Moreover, this finding diverged from the finger tapping task, and is therefore consistent with the idea that task features are an important consideration in determining the relative contribution of each constraint [71], [72], [73].

In summary, we used a versatile task setup to test multiple coordination constraints. We found that as the relative angular velocity between the hands increased, quality of performance decreased, as indicated by changes in finish offset error and absolute deviation. Contrary to finger tapping work, performance of integer ratios was worse than non-integer ratios. We conclude that multiple bimanual constraints can be tested simultaneously within a single visuomotor task framework and such constraints can all be overcome with practice in the presence of augmented visual feedback.
